# LC-MS-Based Metabolomics Reveals the Mechanism of Protection of Berberine against Indomethacin-Induced Gastric Injury in Rats

**DOI:** 10.3390/molecules29051055

**Published:** 2024-02-28

**Authors:** Jing Xu, Xiu-Wei Yang

**Affiliations:** State Key Laboratory of Natural and Biomimetic Drugs, Department of Natural Medicines, School of Pharmaceutical Sciences, Peking University, Beijing 100191, China; xuj2019@bjmu.edu.cn

**Keywords:** berberine, indomethacin-induced, gastric injury, serum metabolomics

## Abstract

Berberine is a natural isoquinoline alkaloid with low toxicity, which exists in a wide variety of medicinal plants. Berberine has been demonstrated to exhibit potent prevention of indomethacin-induced gastric injury (GI) but the related mechanism remains unclear. In the present study, liquid chromatography-mass spectrometry (LC-MS)-based metabolomics was applied for the first time to investigate the alteration of serum metabolites in the protection of berberine against indomethacin-induced gastric injury in rats. Subsequently, bioinformatics was utilized to analyze the potential metabolic pathway of the anti-GI effect of berberine. The pharmacodynamic data indicated that berberine could ameliorate gastric pathological damage, inhibit the level of proinflammatory factors in serum, and increase the level of antioxidant factors in serum. The LC-MS-based metabolomics analysis conducted in this study demonstrated the presence of 57 differential metabolites in the serum of rats with induced GI caused by indomethacin, which was associated with 29 metabolic pathways. Moreover, the study revealed that berberine showed a significant impact on the differential metabolites, with 45 differential metabolites being reported between the model group and the group treated with berberine. The differential metabolites were associated with 24 metabolic pathways, and berberine administration regulated 14 of the 57 differential metabolites, affecting 14 of the 29 metabolic pathways. The primary metabolic pathways affected were glutathione metabolism and arachidonic acid metabolism. Based on the results, it can be concluded that berberine has a gastroprotective effect on the GI. This study is particularly significant since it is the first to elucidate the mechanism of berberine’s action on GI. The results suggest that berberine’s action may be related to energy metabolism, oxidative stress, and inflammation regulation. These findings may pave the way for the development of new therapeutic interventions for the prevention and management of NSAID-induced GI disorders.

## 1. Introduction

Non-steroidal anti-inflammatory drugs (NSAIDs) are the second-largest class of drugs after anti-infective drugs due to their analgesic and anti-inflammatory effects, especially in improving the inflammatory symptoms of rheumatic diseases [1,2]. The frequent use of NSAIDs can cause numerous adverse reactions, including gastric erosions, ulceration, and bleeding [3,4]. In addition, NSAIDs are the second most common etiological factor that destroys the integrity of gastric mucosa after *Helicobacter pylori* infection [5]. The pathophysiological processes that lead to NSAID-induced peptic ulceration are multifactorial and complex. Over the past few decades, several studies have shown that NSAIDs trigger gastric damage by directly impairing the integrity of the stomach mucosa, inhibiting COX-1, reducing the synthesis of prostaglandin, and generating reactive oxygen species [2]. The reactive oxygen species activate inflammatory processes in damaged gastrointestinal tissue [6]. The current prevention strategies for NSAID-associated gastropathy are not very effective [7]. Proton pump inhibitors (PPIs) such as lansoprazole and omeprazole usually require the concomitant administration of H2 blockers or prostaglandin to be more effective [8]. Moreover, although PPIs are effective against gastric injury (GI) to a certain extent, their clinical application is often limited because of side effects [9]. However, there is evidence to suggest that the co-administration of PPIs and NSAIDs may exacerbate intestinal injury and increase the risk of gastric cancer [10,11]. Therefore, complementary and alternative medicine in the GI induced by NSAIDs deserves more attention. Medicinal plant products have been used as possible therapeutic alternatives for gastro-protection over the past few decades and have been the subject of active scientific investigations [12].

Berberine is a natural isoquinoline alkaloid with low toxicity, which exists in a wide variety of medicinal plants [13]. Berberine exhibits several pharmacological activities. Many studies have shown that berberine and its derivatives have high action against digestive system inflammation and cancer [14,15]. At the same time, some articles reported that berberine could reduce the gastric damage caused by NSAIDs by inhibiting oxidative stress and apoptosis [9]. However, the specific pharmacological mechanism is not elucidated clearly.

Metabolites are the end product of cellular regulatory and metabolic processes, and their levels indicate biological responses to environmental changes [16]. Metabolomics is a powerful tool for understanding variations in metabolites due to different processes, revealing the mechanisms involved in changes in endogenous metabolites in different conditions [17,18]. Untargeted metabolomics is a scientific approach that examines alterations in biological endogenous metabolites and identifies metabolic pathways using high-throughput assays. This method is commonly used to comprehend the construction and operation mechanisms of metabolic networks in living organisms like pharmacology, drug toxicology, and the modernization of Chinese medicine [19,20].

**Figure 1:**
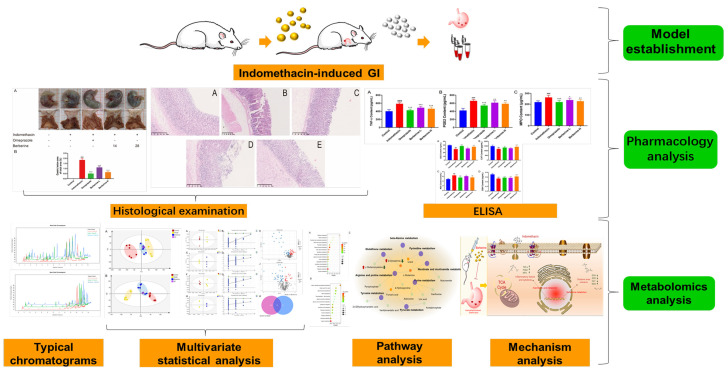
Research design.

In this research, a GI model induced by indomethacin in rats was used to examine the anti-GI impact of berberine. Using LC-MS metabolomics technology, the metabolomics spectrum of berberine was observed for the first time on indomethacin-induced GI in rats (Figure 1). This helped in identifying the metabolic pathway and molecular mechanism of berberine that work against indomethacin-induced GI.

## 2. Results

### 2.1. Effect of Berberine on Gastric Lesions Induced by Indomethacin

**Figure 2:**
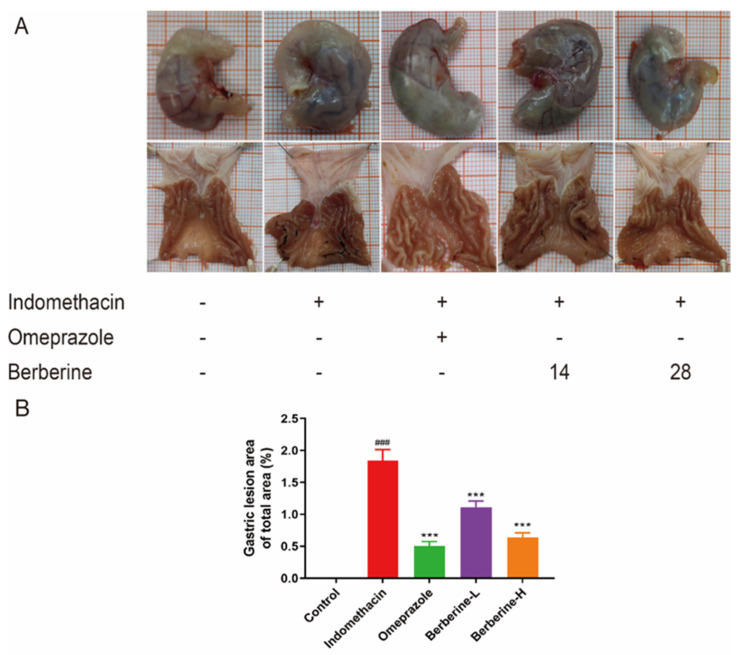
Effect of berberine on (**A**) the appearance of the indomethacin-induced GI in rats and (**B**) the percentage of gastric mucosal lesion area. *n* = 6; ^###^ *p* < 0.001 vs. control group; *** *p* < 0.001 vs. indomethacin group.

**Figure 3:**
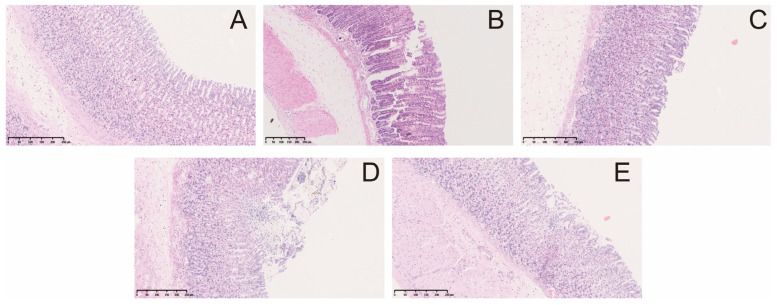
Microscopic pathological effects of berberine on indomethacin-induced gastric injury in rats. control group (**A**), indomethacin group (**B**), omeprazole group (**C**), berberine low-dose group (**D**), and berberine high-dose group (**E**).

Multiple punctate, linear hemorrhagic lesions, submucosal edema, and focal erosions were observed after intragastric injection of indomethacin (Figure 2A). Compared with the model group, the gastric bleeding and erosion of rats in the treatment group were significantly improved. The results of the histopathological examination showed the gastric mucosa tissue structure of normal rats (Figure 3A). In contrast, administration of indomethacin-induced gastric lesions is characterized by loss of gastric epithelial cells and neutrophil infiltration (Figure 3B). Omeprazole and berberine attenuated indomethacin-induced pathological exacerbation (Figure 3C–E). Ulcerated areas in gastric photographs were processed using ImageJ software to characterize the gastrointestinal tracts of rats. Omeprazole and berberine significantly attenuated indomethacin-induced gastrointestinal inflammation (Figure 2B).

### 2.2. Effect of Berberine on Levels of Inflammatory Cytokine Markers in Serum

**Figure 4:**
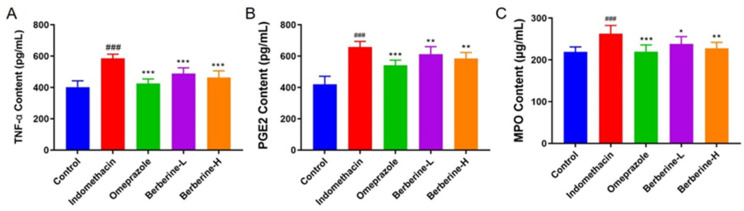
Effect of berberine on the expression of the proinflammatory cytokines in the serum of indomethacin-induced GI in rats. (**A**–**C**) The levels of TNF-α, PGE2, and MPO in rats’ serum. *n* = 6; ^###^ *p* < 0.001 vs. control group; * *p* < 0.05, ** *p* < 0.01, and *** *p* < 0.001 vs. indomethacin group.

The administration of indomethacin significantly (*p* < 0.001) increased the levels of proinflammatory cytokine tumor necrosis factor α (TNF-α), prostaglandin E2 (PGE2), and myeloperoxidase (MPO) in serum compared with the blank group (Figure 4). However, the increase in the three indices described above was notably (*p* < 0.001) decreased by pretreatment with omeprazole or berberine (range from 1.2-fold change to 1.6-fold change), and berberine showed dose-dependent behavior.

### 2.3. Effect of Berberine on Antioxidant Biomarkers in Serum

**Figure 5:**
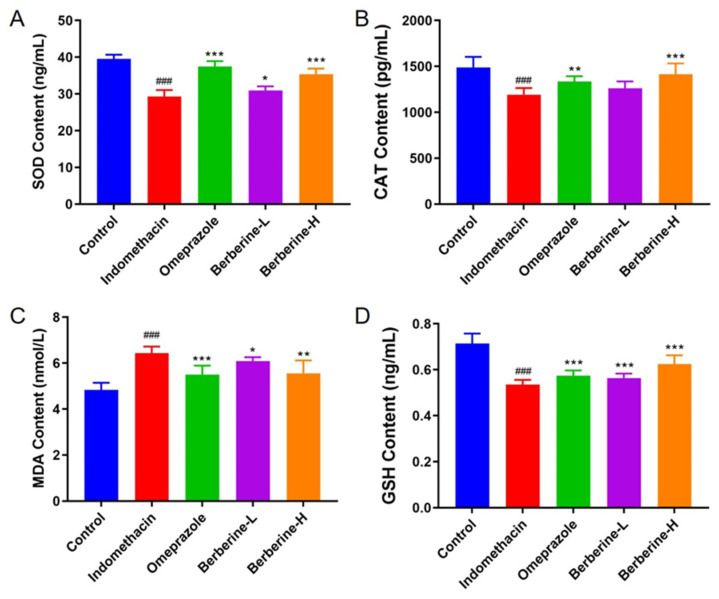
Effect of berberine on antioxidant biomarkers in the serum of indomethacin-induced GI in rats. (**A–D**) The activities/levels of SOD, CAT, GSH, and MDA in rats’ serum. *n* = 6; ^###^ *p* < 0.001 vs. control group; * *p* < 0.05, ** *p* < 0.01, and *** *p* < 0.001 vs. indomethacin group.

The activities of the antioxidant enzymes superoxide dismutase (SOD) and catalase (CAT) and the levels of glutathione peroxidase (GSH) and malondialdehyde (MDA) in gastric tissue of berberine-treated rats are summarized in Figure 5. Rats administered indomethacin showed significant reductions in SOD (25.2% fold change), CAT (16.7% fold change), MDA (20.6% fold change), and GSH (18.7% fold change). The results showed that berberine treatment significantly increased the levels of SOD, CAT, and GSH and decreased the level of MDA. In addition, the therapeutic effect of berberine was dose-dependent. These findings suggest that berberine may have a protective effect against indomethacin-induced oxidative stress in gastric tissue.

### 2.4. Multivariate Statistical Analysis and Potential Biomarkers Exploring

**Figure 6:**
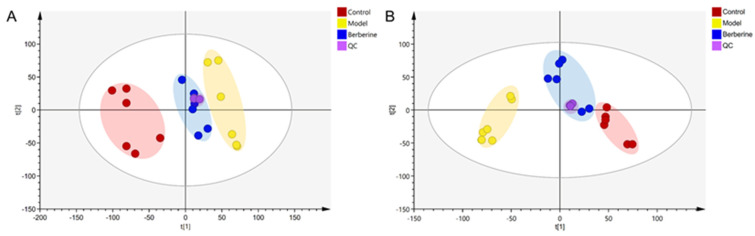
PCA score plot of the control, model, and berberine groups. (**A**): ESI+ model; (**B**): ESI− model.

In this study, using SIMCA-P software (version 14.1), the differences in the serum metabolome between the control group, the model group, and the berberine group were investigated by principal component analysis (PCA). The clustering difference of QCs is slight, which indicates that the instrument has high stability. The clustering of the control, model, and berberine groups was classified in both ESI+ and ESI− modes, suggesting significant differences between these groups (Figure 6A,B).

**Figure 7:**
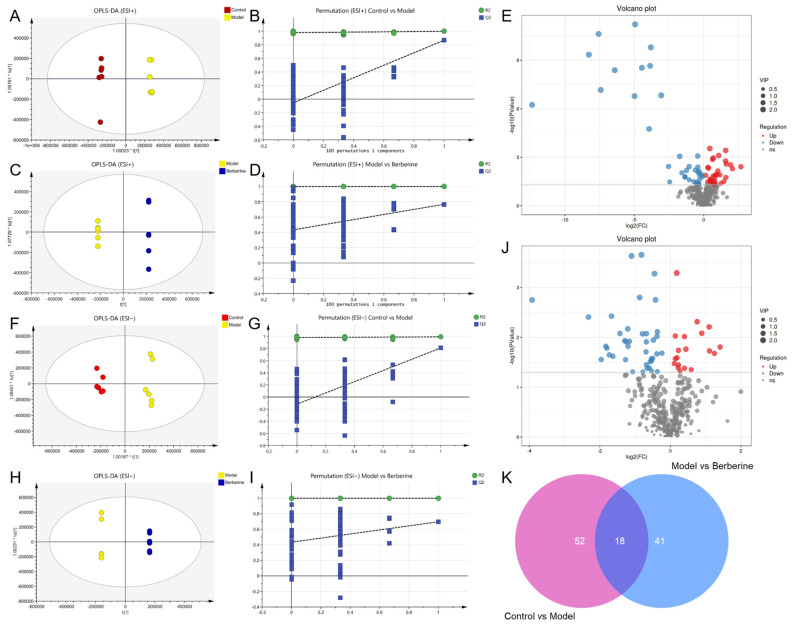
OPLS-DA analysis and potential biomarker exploration of berberine in the treatment of indomethacin-induced GI. (**A**) OPLS-DA score plot of control vs model group in ESI+ mode; (**B**) The 100-permutation test of the OPLS-DA model in ESI+ mode, control vs model group; (**C**) OPLS-DA score plot of model vs berberine group in ESI+ mode; (**D**) The 100-permutation test of the OPLS-DA model in ESI+ mode, model vs berberine group; (**E**) Volcano plots of all serum metabolites detected in ESI+ mode; (**F**) OPLS-DA score plot of control vs model group in ESI− mode; (**G**) The 100-permutation test of the OPLS-DA model in ESI− mode, control vs model group; (**H**) OPLS-DA score plot of model vs berberine group in ESI− mode; (**I**) The 100-permutation test of the OPLS-DA model in ESI− mode, model vs berberine group; (**J**) Volcano plots of all serum metabolites detected in ESI− mode; (**K**) Venn diagram of differential metabolites in control vs model vs berberine group. Pink circle indicates the number of differential metabolites between the control group and the model group. Blue circle indicates the number of differential metabolites between the model group and the berberine group.

Orthogonal partial least squares discriminant analysis (OPLS-DA) was used to distinguish the differences in the metabolites (Figure 7). The R^2^Y and Q^2^ were used to assess the quality of the OPLS-DA models between the control group, the model group, and the berberine group (Table S1). These data indicated that the models are of good fitness and predictability.

### 2.5. Identification and Analysis of Metabolic Biomarkers

Metabolomics is a powerful approach that enables the simultaneous monitoring of multiple metabolic pathways, allowing for the identification of up- or down-regulated marker metabolites and the extrapolation of their underlying regulatory processes. To identify differential metabolites, a *t*-test and OPLS-DA analysis are commonly used, with VIP > 1 and *p* < 0.05 serving as the criteria for significance. By leveraging these analytical tools, researchers can effectively and efficiently identify key metabolic changes [21,22,23]. Following the screening process, it was observed that there existed a total of 70 differential metabolites between the control and model groups, while the model and berberine groups exhibited 59 differential metabolites (Figure 7E,J).

The structure of a compound can be deduced from primary MS results. However, MS/MS is necessary to obtain more precise information and improve the accuracy of the results. To confirm metabolic biomarkers, we conducted searches in databases such as HMDB, MzCloud, LipidMaps, MassBank, and others that rely on MS/MS fragmentation patterns.

According to the above screening conditions, a total of 57 metabolic biomarkers were identified after modeling by indomethacin compared with the control group, including acetylphosphate, 2-hydroxyglutarate, γ-glutamylcysteine, uric acid, indole-3-acetate, etc. (Table S2).

Furthermore, a total of 45 metabolic biomarkers were screened in GI rats treated with berberine, including PGA1, 13S-hydroxyoctadecadienoic acid, prostaglandin F2a, undecanoic acid, and pyrophosphate, etc. (Table S3).

Notably, there were a total of 18 differential metabolites across all three groups, and 14 were identified (Figure 7K). These metabolites are associated with energy metabolism, oxidative stress, and inflammation, and included indoleacetaldehyde, d-Synephrine, corticosterone, estradiol, stearic acid, etc. All of them are summarized in Table 1 with their corresponding compound name, mass (*m*/*z*), formula, −log(*p*), and VIP. Thus, these abovementioned 13 common metabolites were considered to be the potential biomarkers for berberine improving the metabolic changes in indomethacin-induced GI rats. Indomethacin administration in rats increased 5 metabolites and decreased 9. Berberine could alleviate this effect.

### 2.6. Metabolic Pathway Enrichment Analysis

**Figure 8:**
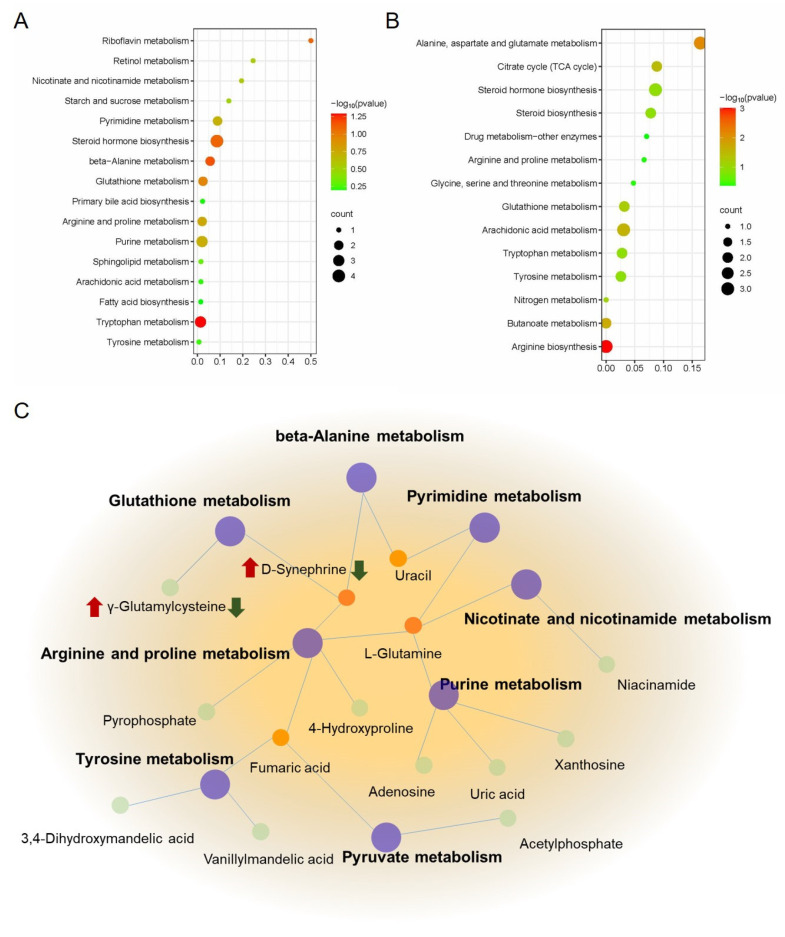
Metabolic pathway analysis of berberine in the treatment of indomethacin-induced GI. (**A**) Metabolic pathway enrichment analysis of control vs model group; (**B**) Metabolic pathway enrichment analysis of model vs berberine group; (**C**) The metabolic network of berberine might affect indomethacin-induced GI. The red mark indicates the change in the relative concentration of metabolites after modeling, and the green mark indicates the change in the relative concentration of metabolites after berberine administration in the model group.

Then, MetaboAnalyst 4.0 was used to construct metabolic pathways for exploring berberine’s mechanism on indomethacin-induced GI, using identified potential metabolites. As shown in Figure 8A, 29 metabolic pathways (Table S4) were enriched in the model group compared with the control group, mainly involving the Tryptophan metabolism, Arachidonic acid metabolism, Primary bile acid biosynthesis, Pantothenate and CoA biosynthesis, and other pathways. As shown in Figure 8B, a total of 24 metabolic pathways (Table S5) were enriched in the GI rats with berberine administration, mainly in arginine biosynthesis, arachidonic acid metabolism, glutathione metabolism, and the citrate cycle (TCA cycle), etc. According to our observations, the model and berberine administration groups have 14 identical metabolic pathways, including arachidonic acid metabolism, butanoate metabolism, tryptophan metabolism, beta-alanine metabolism, steroid hormone biosynthesis, glutathione metabolism, biosynthesis of unsaturated fatty acids, arginine and proline metabolism, purine metabolism, pyrimidine metabolism, nicotinate and nicotinamide metabolism, pyruvate metabolism, cysteine and methionine metabolism, and Tyrosine metabolism. To further understand the metabolic biomarkers and enriched pathways, we mapped a comprehensive metabolic network diagram highlighting the critical interrelationships between pathways. Figure 8C displays this diagram in full detail.

## 3. Discussion

There is mounting evidence to suggest that NSAIDs can instigate the production of proinflammatory mediators and facilitate neutrophil adhesion, thereby jeopardizing the integrity of the mucosa and leading to the development of gastric ulcers [24,25,26]. Simultaneously, NSAIDs can also disturb the equilibrium of endogenous angiogenesis, thereby impeding the process of mucosal repair and healing [12,27,28]. These findings underscore the need for caution in the use of NSAIDs and the importance of monitoring their impact on the gastrointestinal tract. Among NSAIDs, the representative drug indomethacin is considered the main pathogenic factor that induces gastric damage [2]. Therefore, the indomethacin-induced gastric injury model in rats was used to study the gastric protection of berberine. Previous studies have shown that the protective mechanism of berberine against indomethacin gastric injury may involve antioxidant and anti-inflammatory states mediated by the Nrf2 signaling pathway and p38 MAPK translocation [14,15]. However, the elucidation of the precise mechanism is required. We investigated the impact of berberine on indomethacin-induced GI issues in rats by utilizing an LC-MS untargeted metabolomics approach to uncover the possible mechanism for the first time.

The results mentioned above indicate that berberine could effectively alleviate GI distress, reduce the irritation caused by indomethacin on the stomach lining, and minimize the ulcer index. The HE staining observations also confirmed that berberine could significantly reduce gastric tissue hemorrhagic trauma, submucosal oedema, inflammatory cell infiltration, epithelial cell shedding, and other pathological changes, which is consistent with the findings reported in the literature [9]. We conducted an evaluation of the protective effects of berberine against GI using pharmacodynamic indicators. We used low and high doses of berberine to determine its efficacy in preventing indomethacin-induced GI damage. The results showed that the high dose of berberine was more effective in protecting the GI tract. This indicates that berberine has a stronger effect on endogenous metabolites under high-dose conditions. Therefore, the high-dose berberine group was selected for metabonomics analysis to reveal the effect of berberine on endogenous metabolites of indomethacin-induced GI. Subsequently, we will conduct targeted metabolomics experiments to verify the results of untargeted metabolomics and explore the effects of different doses of berberine on metabolites.

In untargeted metabolic projects, two types of chromatographic columns are typically used for detecting different substances. The BEH amide column is ideal for polar metabolites, while the T3 column suits polar metabolites and some strongly polar lipid metabolites [19]. The T3 column has a broader range of applications, so it was chosen for this project.

After conducting a metabolomic analysis, we have identified 14 potential metabolites. This is the first study to report the identification of these biomarkers, which provide a novel insight into the effect of berberine on GI. Compared with the control group, we successfully identified 57 differential metabolites and enriched 29 metabolic pathways by establishing a rat GI model with indomethacin (Table S4). Further, we administered berberine to rats with indomethacin-induced GI and observed 45 differential metabolites and enriched 24 metabolic pathways (as shown in Table S5). Notably, 14 of these pathways were found to be closely associated with the changes observed after indomethacin administration. These metabolites are related to energy metabolism, inflammation, and oxidative stress regulation, suggesting that berberine may affect GI development through these pathways. Among the pathways of shared metabolic compounds, arachidonic acid has been reported to play a key role in the inflammatory process and is associated with GI [29,30]. It is possible to inhibit 12-keto-tetra hydro-leukotriene B4 in the metabolite, which results in the formation of leukotriene B4 (LTB4). LTB4 can attract and activate other white blood cells, such as neutrophils, to sites of inflammation, thereby exacerbating the inflammatory response. It has been previously reported that non-steroidal anti-inflammatory drugs such as indomethacin can cause leukocyte infiltration and increase the level of leukotriene B4 in the stomach, thereby activating inflammatory pathways to attack gastric tissue [31,32,33].

Initially used for the treatment of sepsis and inflammatory bowel disease, γ-glutamylcysteine (γ-GC) acts as an immediate GSH [34]. GSH can effectively scavenge free radicals and other reactive oxygen species (ROS), such as hydroxyl radicals, lipid peroxide radicals, peroxynitrite, and H_2_O_2_. Glutathione is involved in many metabolic processes, including the synthesis of leukotrienes and prostaglandins [35]. Interestingly, berberine increased the level of GSH compared with the model group, indicating that berberine participated in the regulation of the glutathione metabolism signal pathway. These findings are consistent with the effect of berberine on improving oxidative stress.

In addition, this study found that taking indomethacin would affect bile secretion and liver metabolism in rats. It is important to pay attention to the protection of the liver while protecting the stomach. To further verify these results, future research may be required to explore the effects of berberine on genes that are related to inflammation, oxidative stress, and energy metabolism. It is important to pay attention to the collaborative study of liver and stomach simultaneously. Such research may be critical in providing a better understanding of the potential therapeutic benefits of berberine in treating GI issues.

## 4. Materials and Methods

### 4.1. Reagents and Chemicals

Berberine (purity ≥ 98%) was purchased from Xi’an Shengqing Biotechnology Co., Ltd. (Xi’an, China). Omeprazole and indomethacin were purchased from Aladdin Biotechnology Co., Ltd. (Shanghai, China). LC/MS grade methanol, acetonitrile, and formic acid were procured from Fisher Scientific (Fair Lawn, NJ, USA). All other reagents and chemicals used in this study were of analytical grade.

### 4.2. Animals and Indomethacin-Induced GI Model Establishment

Specific pathogen-free male SD rats (200 ± 20 g, SYXK [Jing] 2021-0064) were purchased from the Laboratory Animal Center of Peking University Health Science Center. All animal care and experiments were conducted with the approval of the recommendations of the Peking University Guidelines for the Care and Use of Laboratory Animals (LA2018313) and the National Institutes of Health guidelines.

After precisely tailored feeding for a week, 30 rats were randomly assigned to five groups: a blank control group, an indomethacin (model) group, an omeprazole group (20 mg/kg), and low-dose (14 mg/kg) and high-dose (28 mg/kg) berberine groups [27,36]. The control and indomethacin groups received 0.5% sodium carboxymethylcellulose (CMC-Na) throughout the experiment. All the above drugs were orally administered for 7 days.

After the last dose, all rats were fasted for approximately 24 h with free access to water. Except for the control group, the rats were fed with indomethacin (150 mg/kg) [27,37]. Five hours after indomethacin administration, the rats were anesthetized and dissected. After blood collection, the stomach was quickly obtained and cut along the greater curvature [27]. Then, the whole stomach was douched with clean ice-cold saline. Then, the stomachs of the rats were photographed. The gastric mucosal injury area of each animal was calculated and expressed as a percentage (%) of the total stomach area [38]. After photographing, the gastric tissue was cut and immersed in a 10% neutral buffered formalin solution for histopathological examination. Residual gastric tissues were stored at −80 °C for biochemical analysis.

### 4.3. Histopathological Examination

The fixed tissues were removed from the neutral formalin solution, washed with tap water, dehydrated with different ethanol concentrations, and embedded in paraffin. Subsequently, the gastric tissue was sectioned and then stained with hematoxylin-eosin (HE). Gastric tissues were subjected to histopathological determination by NanoZoomer-SQ pathological slide scanner (Tokyo, Japan).

### 4.4. Measurement of Specific Serum Biomarkers

Enzyme-Linked ImmunoSorbent Assay (ELISA) kits from Beijing Qisong Biotechnology Co., Ltd. (Beijing, China) were used to measure levels of both proinflammatory markers (TNF-α, PGE2, MPO) and antioxidant biomarkers (GSH, CAT, SOD, MDA). The manufacturer’s instructions were adhered to meticulously.

### 4.5. Metabolomics Analysis

#### 4.5.1. Serum Sample Handling

To properly prepare the sample for analysis, a total of 200 µL of serum and 1000 µL of chromatography-grade methanol–acetonitrile were thoroughly combined in a 3:1 ratio. The mixture was then subjected to centrifugation at 10,000× *g* for 10 min at 4 °C to isolate the protein. The resulting supernatant was subsequently dried using a vacuum centrifugal concentrator at 37 °C and 1200 rpm. Following this, the sample was reconstituted with 100 µL of methanol–acetonitrile (3:1) and underwent another round of centrifugation at 10,000× *g* for 10 min at 4 °C. Finally, the supernatant was filtered through a 0.22 µm PTFE microporous membrane to ensure that the preparation was complete and ready for analysis.

#### 4.5.2. QC Samples

To address bias and systematic errors, QC samples were created by pooling 10 μL of each sample from every group and analyzed once for every ten samples.

#### 4.5.3. Chromatography and Mass Spectrometry

The Vanquish UHPLC System from Thermo Fisher Scientific (Waltham, MA, USA) was used for ultra-performance liquid chromatography (UPLC) analysis. A Waters ACQUITY UPLC HSS T3 column (2.1 mm × 100 mm, 1.8 μm) from Waters (Milford, MA, USA) was employed for chromatography and separation. The column was kept at 40 °C, and the flow rate and injection volume were set at 0.3 mL/min and 2 μL, respectively. To conduct LC ESI (+) MS analysis, the mobile phases used were (A) 0.1% formic acid in water (*v*/*v*) and (B) 0.1% formic acid in acetonitrile (*v*/*v*). For LC ESI (−) MS analysis, the analytes were carried out with (A) ammonium formate (5 mM) and (B) acetonitrile. Please refer to Table 2 for information on the gradient elution procedures.

We utilized mass spectrometry on an Orbitrap Exploris120 (ThermoFisher Scientific, USA) with an ESI ion source to detect metabolites. The acquisition method employed was simultaneous MS1 and MS/MS (Full MS ddMS2 mode, data-dependent MS/MS), with the following parameters: sheath gas pressure at 40 arb, auxiliary gas flow at 10 arb, spray voltage at 3.50 kV and 2.50 kV for ESI (+) and ESI (−), respectively, capillary temperature at 325 °C, MS1 range at *m*/*z* 100–1000 Da, MS1 resolving power at 60,000 FWHM, number of data-dependent scans per cycle at 4, MS/MS resolving power at 15,000 FWHM, normalized collision energy at 30%, and automatic dynamic exclusion time. The typical chromatogram is shown in Figure S1.

#### 4.5.4. Data Processing and Multivariate Analysis

To convert the original mass spectrum files, the Proteowizard software package (v3.0.8789) was used to change them to the mzXML file format [39]. The R XCMS (v3.12.0) software package was used for peak detection, peak filtering, and peak alignment processing [40]. To eliminate systematic errors, support vector regression correction was applied based on QC samples. The processed data were then imported into the SIMCA-P software (version14.1; Umetrics, Umea, Sweden) for multivariate statistical analysis, which included PCA and OPLS-DA. PCA is a widely used statistical method for examining the correlation between multiple variables. By analyzing the correlation structure of the data, it can reveal the internal structure of multiple variables through a few principal components. PCA is capable of visually reflecting the overall distribution characteristics of all samples and trends [19]. OPLS-DA is based on partial least squares discriminant analysis (PLS-DA), which improves the interpretability and accuracy of multivariate discriminant analysis. It is commonly used to filter out significantly different metabolites in two groups [30].

#### 4.5.5. Potential Biomarker Identification and Pathway Enrichment Analysis

The biomarkers that met the conditions of *p*-value less than 0.05 and VIP score greater than 1 in the multivariate analysis were selected for further evaluation [17,41,42]. Accurate mass-to-charge ratios were based on differential metabolites, within a permissible range of matched mass deviations of 30 ppm. To validate the data, we compared the mass spectrometry fragment ions and isotopes with various online metabolomics databases, including HMDB (http://www.hmdb.ca/, accessed on 1 December 2023), METLIN (http://metlin.scripps.edu/, accessed on 1 December 2023) [43], massbank, and LipidMaps [19]. This was accomplished by performing data validation, which involved a detailed examination of the aforementioned databases to ensure consistency between the metabolites under investigation and their corresponding entries in the databases.

#### 4.5.6. Pathway Analysis

Functional pathway enrichment and topological analysis were conducted using MetaboAnalyst 4.0 to screen for metabolic biomarkers. Pathways were visualized using the KEGG Mapper tool.

### 4.6. Statistical Analysis

The data underwent analysis via the one-way analysis of variance (ANOVA) alongside the Bonferroni method. A *p*-value lower than 0.05 was regarded as statistically significant. The outcomes were displayed as the mean value with standard deviation. ImageJ software was utilized for the grayscale analysis, while GraphPad Prism 9 software was employed to visualize all the results.

## 5. Conclusions

**Figure 9:**
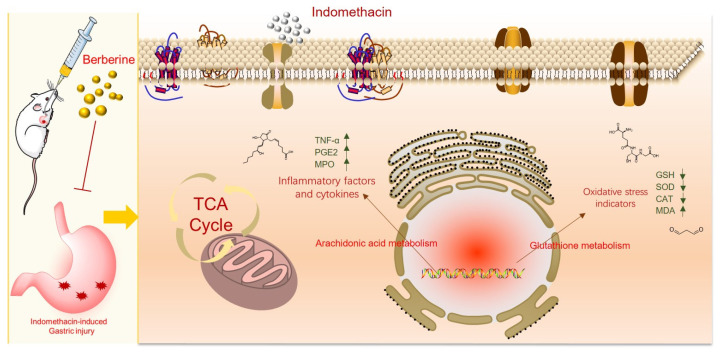
The mechanism of berberine in treating indomethacin-induced GI.

Our findings unequivocally demonstrate that berberine treatment efficiently mitigates gastric mucosal injury in rats afflicted with indomethacin-induced GI problems The present study utilized serum metabolomics to investigate the underlying mechanism of berberine against gastrointestinal issues. Remarkably, we identified 14 endogenous metabolites in serum that served as biomarkers to explicate berberine’s mechanism of action. This is the first study to report the identification of these biomarkers, which provide a novel insight into the effect of berberine on GI. Our research has uncovered 14 metabolic pathways of berberine that may be effective in treating GI in rats induced by indomethacin. The crucial mechanism of berberine in treating indomethacin-induced GI is shown in Figure 8C and Figure 9. Berberine has been shown to have a complex mechanism of action on the GI. This mechanism may involve the regulation of energy metabolism, oxidative stress, and inflammation. The implications of these findings are twofold. Firstly, they offer valuable insight into the treatment of GI issues induced by indomethacin, an NSAID drug. Secondly, they shed light on a possible mechanism of action of berberine for treating GI problems. According to the study, berberine can be a promising drug for treating GI disorders caused by NSAIDs. Moreover, the identification of metabolites and pathways can be potential drug targets for diagnosing and treating GI disorders. Further research is required to investigate the role of berberine in regulating endogenous metabolites associated with NSAID-induced GI issues. These findings may lead to the development of new therapeutic interventions for the prevention and management of NSAID-induced GI disorders. 

## Figures and Tables

**Table 1:** Differential metabolites identified in the serum of the control, model, and berberine groups.

No.	Metabolism	*m*/*z*	Formula	Changed Trend	
				Model/Control	Model/Berberine
1	d-Synephrine	168.0918	C_9_H_13_NO_2_	Up ^#^	Down **
2	l-2-Amino-6-oxoheptanedioate	190.0863	C_7_H_11_NO_5_	Up ^###^	Down *
3	Butyryl-l-carnitine	232.1549	C_11_H_21_NO_4_	Up ^###^	Down *
4	Estradiol	273.2536	C_18_H_24_O_2_	Down ^#^	Up *
5	Dehydroepiandrosterone	288.2896	C_19_H_28_O_2_	Down ^#^	Up *
6	Epipregnanolone	319.2681	C_21_H_34_O_2_	Down ^#^	Up *
7	12-Keto-tetrahydro-leukotriene B4	336.3267	C_20_H_32_O_4_	Down ^##^	Up **
8	(*R*)-3-Hydroxybutyric acid	103.0397	C_4_H_8_O_3_	Down ^##^	Up *
9	3-Methylthiopropionic acid	118.9269	C_4_H_8_O_2_S	Up ^#^	Down *
10	Dodecanedioic acid	229.1447	C_12_H_22_O_4_	Down ^##^	Up *
11	γ-Glutamylcysteine	248.9601	C_8_H_14_N_2_O_5_S	Up ^###^	Down **
12	Stearic acid	283.2605	C_18_H_36_O_2_	Down ^#^	Up *
13	Corticosterone	345.2060	C_21_H_30_O_4_	Down ^#^	Up *
14	Indoleacetaldehyde	160.0758	C_10_H_9_NO	Down ^#^	Up *

Note: ^#^ *p* < 0.05, ^##^ *p* < 0.01, and ^###^ *p* < 0.001 vs. control group; * *p* < 0.05, ** *p* < 0.01 vs. indomethacin group.

**Table 2:** Gradient elution procedures.

Time (min)	Positive Mode		Negative Mode	
	A (%)	B (%)	A (%)	B (%)
0–1	92	8	92	8
1–8	92–2	8–98	92–2	89–8
8–10	2	98	2	98
10–10.1	29–2	98–8	2–92	98–8
10.1–12	92	8	92	8

## Data Availability

The original contributions presented in the study are included in the article/Supplementary Material. Further inquiries can be directed to the corresponding authors.

## Supplementary Materials

The following supporting information can be downloaded at: https://www.mdpi.com/article/10.3390/molecules29051055/s1, Figure S1: Typical chromatogram; Table S1: R^2^Y and Q^2^ results in control vs model and model vs berberine under ESI+/− mode; Table S2: Metabolites identified in serum of control vs model; Table S3: Metabolites identified in serum of model vs berberine; Table S4: Results of KEGG enrichment pathway in control group and model group; Table S5: Results of KEGG enrichment pathway in model group and berberine group.
