# Supplementary material for: LC-MS-Based Metabolomics Reveals the Mechanism of Protection of Berberine against Indomethacin-Induced Gastric Injury in Rats
Source: Molecules. 2024 Feb 28;29(5):1055. doi: 10.3390/molecules29051055 (PMC10934493; doi:10.3390/molecules29051055)
Supplement: Supplementary file 1 [file molecules-29-01055-s001.zip › Table S1.pdf]

Table S1. R^2Y and Q^2 results in control *vs* model and model *vs* berberine under ESI+/- mode

	ESI+		ESI-	
	control *vs* model	model *vs* berberine	control *vs* model	model *vs* berberine
R^2Y (cum)	0.999	0.999	1	0.999
Q^2 (cum)	0.754	0.737	0.799	0.773
