# Supplementary material for: LC-MS-Based Metabolomics Reveals the Mechanism of Protection of Berberine against Indomethacin-Induced Gastric Injury in Rats
Source: Molecules. 2024 Feb 28;29(5):1055. doi: 10.3390/molecules29051055 (PMC10934493; doi:10.3390/molecules29051055)
Supplement: Supplementary file 1 [file molecules-29-01055-s001.zip › Table S2.pdf]

Table S2. Metabolites identified in serum of control *vs* model

No.	Compound name	*m/z*	Formula	-log10 P	VIP	Ion
1	3-Methylthiopropanamine	106.0653	$C_4H_{11}NS$	1.5	1.490421	+
2	Hydroquinone	110.0208	$C_6H_6O_2$	1.36	1.41648	+
3	Phosphonoacetate	123.0806	$C_2H_5O_5P$	1.33	1.382809	+
4	Niacinamide	123.0556	$C_6H_6N_2O$	2.77	1.929041	+
5	Isoquinoline	130.0654	C_9H_7N	1.76	1.757412	+
6	L-Pipecolic acid	130.0864	$C_6H_{11}NO_2$	3.11	2.024051	+
7	6-Hydroxyhexanoic acid	132.0791	$C_6H_{12}O_3$	1.62	1.541846	+
8	*cis*-4-Hydroxy-D-proline	132.1021	$C_5H_9NO_3$	2.33	1.970043	+
9	L-Leucine	132.102	$C_6H_{13}NO_2$	1.84	1.622443	+
10	2-Phenylacetamide	136.0758	C_8H_9NO	3.08	2.003445	+
11	Acetylphosphate	140.9925	$C_2H_5O_5P$	7.16	2.407867	+
12	8-Hydroxyquinoline	146.0603	C_9H_7NO	1.52	1.534747	+
13	2-Hydroxyglutarate	148.0432	$C_5H_8O_5$	2.04	1.717831	+
14	*trans*-Cinnamate	148.0427	$C_9H_8O_2$	1.61	1.51122	+
15	Imidazol-5-yl-pyruvate	155.0469	$C_6H_6N_2O_3$	2.59	1.875173	+
16	4,5-Dihydroorotic acid	158.9594	$C_5H_6N_2O_4$	2.53	1.8555	+
17	Indoleacetaldehyde	160.0758	$C_{10}H_9NO$	1.94	1.86245	+
18	D-synephrine	168.0918	$C_9H_{13}NO_2$	1.91	1.664155	+
19	Uric acid	169.0356	$C_5H_4N_4O_3$	1.65	1.590082	+
20	Amino acid(Arg-)	175.1193	$C_6H_{14}N_4O_2$	1.48	1.473219	+
21	Indole-3-acetate	176.0709	$C_{10}H_9NO_2$	9.8	2.39836	+
22	3,4-Dihydroxymandelic acid	184.1696	$C_8H_8O_5$	2.87	1.984189	+
23	N-Alpha-acetyllysine	188.0706	$C_8H_{16}N_2O_3$	1.44	1.491138	+
24	Azelaic acid	188.1283	$C_9H_{16}O_4$	1.39	1.414702	+
25	L-2-Amino-6-oxoheptanedioate	190.0863	$C_7H_{11}NO_5$	6.83	2.40054	+
26	Diaminopimelic acid	191.0406	$C_7H_{14}N_2O_4$	11.22	2.404973	+
27	Spermine	203.1393	$C_{10}H_{26}N_4$	1.35	1.421788	+
28	5-Methoxyindoleacetate	206.0812	$C_{11}H_{11}NO_3$	8.54	2.384973	+
29	Butyryl-L-carnitine	232.1549	$C_{11}H_{21}NO_4$	4.76	2.235437	+
30	Farnesoic acid	236.1624	$C_{15}H_{24}O_2$	3.54	2.101893	+
31	Adenosine	250.094	$C_{10}H_{13}N_5O_4$	8.38	2.38031	+
32	17a-Estradiol	273.1899	$C_{18}H_{24}O_2$	2.21	1.75368	+
33	Estradiol	273.2536	$C_{18}H_{24}O_2$	1.88	1.673566	+
34	Retinal	285.222	$C_{20}H_{28}O$	1.77	1.635077	+
35	Dehydroepiandrosterone	288.2896	$C_{19}H_{28}O_2$	1.51	1.483484	+
36	Epipregnanolone	319.2681	$C_{21}H_{34}O_2$	1.75	1.640052	+
37	Ethyl icosapentate	331.2638	$C_{22}H_{34}O_2$	1.52	1.486911	+
38	12-Keto-tetrahydro-leukotriene B4	336.3267	$C_{20}H_{32}O_4$	2.96	1.98499	+
39	Riboflavin	376.2587	$C_{17}H_{20}N_4O_6$	2.19	1.757357	+
40	(*R*)-3-Hydroxybutyric acid	103.0397	$C_4H_8O_3$	2.16	1.716631	-
41	Uracil	111.0202	$C_4H_4N_2O_2$	6.78	2.339128	-
42	L-Norvaline	116.0717	$C_5H_{11}NO_2$	2.13	1.700603	-
43	3-Methylthiopropionic acid	118.9269	$C_4H_8O_2S$	1.48	1.443557	-
44	4-Hydroxyproline	130.0511	$C_5H_9NO_3$	2.42	1.920154	-
45	Leucine	130.0873	$C_6H_{13}NO_2$	2.08	1.689874	-
46	Phenyllactate	165.0555	$C_9H_{10}O_3$	1.8	1.594277	-
47	Dodecanedioic acid	229.1447	$C_{12}H_{22}O_4$	2.42	1.791389	-
48	gamma-Glutamylcysteine	248.9601	$C_8H_{14}N_2O_5S$	10.63	2.337868	-

49	Palmitic acid	255.2327	$C_{16}H_{32}O_2$	1.33	1.491543	-
50	Glucose 6-phosphate	259.0215	$C_6H_{13}O_9P$	1.79	1.585735	-
51	Xanthosine	283.0675	$C_{10}H_{12}N_4O_6$	8.65	2.321757	-
52	Stearic acid	283.2605	$C_{18}H_{36}O_2$	1.62	1.51961	-
53	Hesperetin	301.2167	$C_{16}H_{14}O_6$	1.48	1.449451	-
54	11-Dehydrocorticosterone	325.1842	$C_{21}H_{28}O_4$	1.93	1.633819	-
55	Corticosterone	345.206	$C_{21}H_{30}O_4$	1.51	1.590411	-
56	Sphingosine 1-phosphate	378.2396	$C_{18}H_{38}NO_5P$	1.47	1.427837	-
57	Glycocholic acid	464.2993	$C_{26}H_{43}NO_6$	1.91	1.800592	-
