# Supplementary material for: LC-MS-Based Metabolomics Reveals the Mechanism of Protection of Berberine against Indomethacin-Induced Gastric Injury in Rats
Source: Molecules. 2024 Feb 28;29(5):1055. doi: 10.3390/molecules29051055 (PMC10934493; doi:10.3390/molecules29051055)
Supplement: Supplementary file 1 [file molecules-29-01055-s001.zip › Table S3.pdf]

Table S3. Metabolites identified in serum of model *vs* berberine

No.	Compound name	*m/z*	Formula	-log10 P	VIP	Ion
1	Creatinine	113.9626	$C_4H_7N_3O$	2.75	2.049786	+
2	Fumaric acid	116.1073	$C_4H_4O_4$	2.07	1.816619	+
3	Nicotinic acid	124.0864	$C_6H_5NO_2$	1.58	1.604297	+
4	*cis*-4-Hydroxy-L-proline	130.9678	$C_5H_9NO_3$	1.73	1.663875	+
5	5,6-Dihydro-5-fluorouracil	133.0319	$C_4H_5FN_2O_2$	1.35	1.476003	+
6	L-Glutamine	146.0814	$C_5H_{10}N_2O_3$	1.32	1.487901	+
7	Indoleacetaldehyde	160.0758	$C_{10}H_9NO$	1.76	1.797117	+
8	Glucosamine	162.0749	$C_6H_{13}NO_5$	1.54	1.587916	+
9	D-synephrine	168.0918	$C_9H_{13}NO_2$	2.02	1.773734	+
10	(*R*)-4-Hydroxymandelate	169.0587	$C_8H_8O_4$	2.08	1.814262	+
11	2-Isopropylmalic acid	176.0711	$C_7H_{12}O_5$	1.45	1.559214	+
12	Undecanoic acid	186.9538	$C_{11}H_{22}O_2$	2.43	1.948965	+
13	Homo-L-arginine	188.1282	$C_7H_{16}N_4O_2$	2.31	1.906716	+
14	L-2-Amino-6-oxoheptanedioate	190.0863	$C_7H_{11}NO_5$	1.67	1.793575	+
15	5-Hydroxyindoleacetic acid	192.0662	$C_{10}H_9NO_3$	1.57	1.58251	+
16	Propionylcarnitine	218.139	$C_{10}H_{19}NO_4$	1.32	1.441066	+
17	Butyryl-L-carnitine	232.1549	$C_{11}H_{21}NO_4$	1.55	1.612011	+
18	Estradiol	273.2536	$C_{18}H_{24}O_2$	1.94	1.903025	+
19	Dehydroepiandrosterone	288.2896	$C_{19}H_{28}O_2$	1.79	1.694491	+
20	Epipregnanolone	319.2681	$C_{21}H_{34}O_2$	1.82	1.749362	+
21	11,12-DiHETrE	321.2416	$C_{20}H_{34}O_4$	2.11	1.818469	+
22	12-Keto-tetrahydro-leukotriene B4	336.3267	$C_{20}H_{32}O_4$	2.02	1.819326	+
23	Prostaglandin F2a	355.2814	$C_{20}H_{34}O_5$	2.21	1.843128	+
24	Deoxycholic acid	375.2881	$C_{24}H_{40}O_4$	1.38	1.627083	+
25	Zymosterol intermediate 2	384.345	$C_{27}H_{44}O$	1.69	1.691494	+
26	3alpha,7alpha,12beta-Trihydroxy-5beta-cholanate	391.2837	$C_{24}H_{40}O_5$	2.41	1.921283	+
27	Ursodeoxycholic acid	393.2092	$C_{24}H_{40}O_4$	3.66	2.206782	+
28	beta-Sitosterol	397.3786	$C_{29}H_{50}O$	1.51	1.582752	+
29	Sodium deoxycholate	415.2104	$C_{24}H_{39}O_4.Na$	2.75	2.030404	+
30	Avermectin A1b aglycone	567.3192	$C_{34}H_{48}O_8$	1.82	1.721925	+
31	(*R*)-3-Hydroxybutyric acid	103.0397	$C_4H_8O_3$	1.39	1.49997	-
32	Betaine	116.9277	$C_5H_{11}NO_2$	1.74	1.651722	-
33	3-Methylthiopropionic acid	118.9269	$C_4H_8O_2S$	1.56	1.694171	-
34	Spermidine	143.9155	$C_7H_{19}N_3$	1.33	1.409259	-
35	Oxoglutaric acid	145.0144	$C_5H_6O_5$	1.45	1.533445	-
36	Pyrophosphate	176.9358	$H_4P_2O_7$	3.28	2.108975	-
37	Vanillylmandelic acid	197.0429	$C_9H_{10}O_5$	1.81	1.662369	-
38	Dodecanedioic acid	229.1447	$C_{12}H_{22}O_4$	1.62	1.74135	-
39	gamma-Glutamylcysteine	248.9601	$C_8H_{14}N_2O_5S$	2.08	1.780825	-
40	Stearic acid	283.2605	$C_{18}H_{36}O_2$	1.93	1.875388	-
41	Epiandrosterone	288.9551	$C_{19}H_{30}O_2$	1.71	1.637566	-
42	13S-hydroxyoctadecadienoic acid	295.2275	$C_{18}H_{32}O_3$	1.48	1.522462	-
43	PGA1	335.2226	$C_{20}H_{32}O_4$	1.31	1.4195	-
44	Corticosterone	345.206	$C_{21}H_{30}O_4$	1.91	1.718393	-
45	3alpha,7alpha-Dihydroxy-12-oxo-5beta-cholanate	405.2648	$C_{24}H_{38}O_5$	2.07	1.801474	-
