# Supplementary material for: LC-MS-Based Metabolomics Reveals the Mechanism of Protection of Berberine against Indomethacin-Induced Gastric Injury in Rats
Source: Molecules. 2024 Feb 28;29(5):1055. doi: 10.3390/molecules29051055 (PMC10934493; doi:10.3390/molecules29051055)
Supplement: Supplementary file 1 [file molecules-29-01055-s001.zip › Table S4.pdf]

Table S4. Results of KEGG enrichment pathway in control group and model group

| No. | Pathway name | Total | Hits | *P* | Impact |
|---|---|---|---|---|---|
| 1 | Butanoate metabolism | 15 | 2 | 0.03747 | 0.0000 |
| 2 | Neomycin, kanamycin and gentamicin biosynthesis | 2 | 1 | 0.04135 | 0.0000 |
| 3 | Tryptophan metabolism | 41 | 3 | 0.05155 | 0.0139 |
| 4 | beta-Alanine metabolism | 21 | 2 | 0.06936 | 0.0560 |
| 5 | Riboflavin metabolism | 4 | 1 | 0.08105 | 0.5000 |
| 6 | Steroid hormone biosynthesis | 80 | 4 | 0.08153 | 0.0861 |
| 7 | Glutathione metabolism | 28 | 2 | 0.11415 | 0.0246 |
| 8 | Valine, leucine and isoleucine biosynthesis | 8 | 1 | 0.15571 | 0.0000 |
| 9 | Biosynthesis of unsaturated fatty acids | 36 | 2 | 0.17193 | 0.0000 |
| 10 | Arginine and proline metabolism | 36 | 2 | 0.17193 | 0.0209 |
| 11 | Purine metabolism | 71 | 3 | 0.18223 | 0.0205 |
| 12 | Pyrimidine metabolism | 39 | 2 | 0.19475 | 0.0889 |
| 13 | D-Amino acid metabolism | 15 | 1 | 0.27247 | 0.0000 |
| 14 | Nicotinate and nicotinamide metabolism | 15 | 1 | 0.27247 | 0.1943 |
| 15 | Retinol metabolism | 16 | 1 | 0.28782 | 0.2455 |
| 16 | Starch and sucrose metabolism | 18 | 1 | 0.31758 | 0.1385 |
| 17 | Pantothenate and CoA biosynthesis | 20 | 1 | 0.34613 | 0.0000 |
| 18 | Pyruvate metabolism | 23 | 1 | 0.38680 | 0.0000 |
| 19 | Lysine degradation | 30 | 1 | 0.47239 | 0.0000 |
| 20 | Inositol phosphate metabolism | 30 | 1 | 0.47239 | 0.0000 |
| 21 | Sphingolipid metabolism | 32 | 1 | 0.49464 | 0.0156 |
| 22 | Cysteine and methionine metabolism | 33 | 1 | 0.50542 | 0.0000 |
| 23 | Fatty acid elongation | 39 | 1 | 0.56558 | 0.0000 |
| 24 | Fatty acid degradation | 39 | 1 | 0.56558 | 0.0000 |

| | | | | | |
|---|---|---|---|---|---|
| 25 | Valine, leucine and isoleucine degradation | 40 | 1 | 0.57489 | 0.0000 |
| 26 | Tyrosine metabolism | 42 | 1 | 0.59294 | 0.0067 |
| 27 | Arachidonic acid metabolism | 44 | 1 | 0.61024 | 0.0153 |
| 28 | Primary bile acid biosynthesis | 46 | 1 | 0.62683 | 0.0229 |
| 29 | Fatty acid biosynthesis | 47 | 1 | 0.63487 | 0.0147 |
