# Supplementary material for: LC-MS-Based Metabolomics Reveals the Mechanism of Protection of Berberine against Indomethacin-Induced Gastric Injury in Rats
Source: Molecules. 2024 Feb 28;29(5):1055. doi: 10.3390/molecules29051055 (PMC10934493; doi:10.3390/molecules29051055)
Supplement: Supplementary file 1 [file molecules-29-01055-s001.zip › Table S5.pdf]

Table S5. Results of KEGG enrichment pathway in model group and berberine group

No.	pathway_name	Total	Hits	*P*	Impact
1	Arginine biosynthesis	14	3	0.00097	0
2	Alanine, aspartate and glutamate metabolism	28	3	0.00759	0.16426
3	Butanoate metabolism	15	2	0.02011	0
4	Arachidonic acid metabolism	44	3	0.02621	0.03056
5	Citrate cycle (TCA cycle)	20	2	0.03477	0.08837
6	Glutathione metabolism	28	2	0.06435	0.03179
7	Nitrogen metabolism	6	1	0.08690	0
8	Steroid hormone biosynthesis	80	3	0.11456	0.08607
9	Tryptophan metabolism	41	2	0.12421	0.0278
10	Steroid biosynthesis	41	2	0.12421	0.07785
11	Tyrosine metabolism	42	2	0.12927	0.02578
12	Nicotinate and nicotinamide metabolism	15	1	0.20384	0
13	beta-Alanine metabolism	21	1	0.27369	0
14	Pyruvate metabolism	23	1	0.29564	0
15	Lipoic acid metabolism	28	1	0.34777	0
16	Glyoxylate and dicarboxylate metabolism	32	1	0.38680	0
17	Cysteine and methionine metabolism	33	1	0.39620	0
18	Glycine, serine and threonine metabolism	34	1	0.40547	0.04766
19	Biosynthesis of unsaturated fatty acids	36	1	0.42359	0
20	Arginine and proline metabolism	36	1	0.42359	0.06628
21	Drug metabolism - other enzymes	38	1	0.44118	0.07059
22	Pyrimidine metabolism	39	1	0.44979	0
23	Amino sugar and nucleotide sugar metabolism	42	1	0.47484	0
24	Purine metabolism	71	1	0.66697	0
